# Recent Advances in Medical Therapy for Urological Cancers

**DOI:** 10.3389/fonc.2022.746922

**Published:** 2022-04-04

**Authors:** Takeshi Yuasa, Tetsuya Urasaki, Ryosuke Oki

**Affiliations:** ^1^ Department of Urology, Cancer Institute Hospital, Japanese Foundation for Cancer Research, Tokyo, Japan; ^2^ Department of Medical Oncology, Cancer Institute Hospital, Japanese Foundation for Cancer Research, Tokyo, Japan

**Keywords:** immune checkpoint inhibitor, PARP inhibitor, olaparib, antibody-drug conjugate, androgen receptor axis targeted agent, enfortumab vedotin

## Abstract

The mainstay of medical treatment has been tyrosine kinase inhibitors (TKIs) for renal cell cancer (RCC), cytotoxic chemotherapy for urothelial cancer (UC), and androgen deprivation therapy for prostate cancer. These therapeutic modalities still play important roles in these malignancies. However, immune checkpoint inhibitors (ICIs) that target PD-1/PD-L1 or CTLA-4 are being rapidly introduced for the treatment of metastatic urological cancers, just as they have been for other malignancies. Currently, the paradigm of medical treatment for patients with metastatic urological cancer is dramatically changing. Accordingly, we need to organize and summarize the new therapeutic tools, which include immune checkpoint inhibitors, poly (ADP-ribose) polymerase (PARP) inhibitors, and antibody-drug conjugates (ADCs). This review provides an overview of agents and regimens that have just launched or will be launched in the near future in Japan. Based on the promising anti-tumor efficacy and manageable safety profiles being demonstrated in clinical trials, these new agents and therapies are expected to be rapidly introduced in Japanese clinical practice. Additionally, the newly designed ADC, enfortumab vedotin, which comprises a fully human monoclonal antibody conjugated to an anti-cancerous agent *via* a protease-cleavable linker, has just been launched in Japan. In order to provide the optimal treatment for our patients, we need to completely understand these new therapeutic tools.

## Introduction

Tyrosine kinase inhibitors (TKIs), cytotoxic chemotherapy, and androgen deprivation therapy (ADT) have been the mainstay of medical therapy for metastatic renal cell cancer (RCC), urothelial cancer (UC), and prostate cancer (PC), respectively (1–3). These therapeutic modalities still play important roles in these respective malignancies. However, immune checkpoint inhibitors (ICIs), which target signaling through programmed death-1 (PD-1, which is expressed on activated T cells), PD-ligand 1 and 2 (PD-L1 and PD-L2, which are expressed on antigen-presenting cells [APC] and cancer cells), and cytotoxic T-lymphocyte(associated)antigen-4 (CTLA-4) have been rapidly introduced for the treatment of metastatic urological cancers just as they have been for other malignancies (2–5). In addition, olaparib (Lynparza, AstraZeneca), a poly (ADP-ribose) polymerase (PARP) inhibitor, was just approved for the treatment of castration-resistant prostate cancer (CRPC) harboring BRCA1/2 mutations (6, 7). Moreover, enfortumab vedotin (Padcev, Astellas), a newly designed antibody-drug conjugate (ADC) comprising a fully human monoclonal antibody against a tumor-associated antigen conjugated to an anti-cancer agent *via* a protease-cleavable linker, has been just launched in Japanese clinical practice (8). The paradigm of the medical treatment for patients with metastatic urological cancer is and will be dramatically changing. In this article, we provide a brief overview of these novel agents and a comprehensive summary of the medical treatment of urological cancers, including ongoing clinical trials. These agents are expected to be successfully introduced in Japanese clinical practice soon (**Table 1**).

**Table 1:** Results of the clinical trials of the medical treatment for the urological cancers.

Trial	Therapeutic line	Treatment	Cancer type	Patients	Primary endpoint/Result	Secondary endpoint/Result
**Renal cell cancer**						
Checkmate-214 (9)	1st line	Nivolumab + Ipilimumab vs. Sunitinib	ccRCC	n=1096 (554/546) IMDC risk score Fav. 125/124 Int. 334/333 Poor. 91/89	Coprimary endpoint: OS, ORR, PFS in IMDC int. or poor risk OS: NR vs 26.0 m (HR 0.63, p<0.001) ORR: 42% vs. 27% (p<0.001) PFS: 11.6m vs 8.4m (HR 0.83, p=0.03)	OS, PFS, ORR in ITT population OS: NR vs. 32.9m (HR 0.68, p<0.001) ORR: 39% vs 32 (p=0.02, not significant) PFS: 12.4m vs. 12.3m (HR 0.98, p=0.85)
Javelin Renal 101 (10)	1st line	Pembrolizumab + Avelumab vs. Sunitinib	ccRCC	n=886 (442/444) PD-L1+: (n=560)	PFS, OS with PD-L1 positive tumors OS: immature data PFS: 13.8m vs. 7.2m (HR 0.61, p<0.001)	PFS in overall population: 13.8m vs. 8.4m (HR 0.69, p<0.001) ORR with PD-L1 positive tumors: 55.2% vs. 25.5%
Keynote-426 (11)	1st line	Pembrolizumab + Axitinib vs. Sunitinib	ccRCC	n=861 (432/429)	OS, PFS in ITT population OS: NR vs. NR (HR 0.53, p<0.0001) PFS: 15.1m vs 11.1m (HR 0.69, p<0.001)	ORR: 59.3% vs. 35.7% (p<0.001)
METEOR trial (12)	2nd line	Cabozantinib vs. Everolimus	ccRCC	n=658 (330/328)	PFS: 7.4m vs. 3.8m (HR 0.58, p<0.001)	OS: NR vs. NR (HR 0.67, p=0.005) ORR: 21% vs. 5% (p<0.001)
CABOSUN trial (13)	1st line (Phase2)	Cabozantinib vs. Sunitinib	ccRCC	n=157 (79/78) IMDC risk score: int. or poor	PFS: 8.2m vs. 5.6m (HR 0.66, p=0.012)	OS: 30.3m vs. 21.8m (Adjusted HR 0.80) ORR: 46% vs. 18%
Checkmate 9ER (14)	1st line	Cabozantinib + Nivolumab vs. Sunitinib	ccRCC	n=631 (323/328)	PFS: 16.6m vs 8.3m (HR 0.51, p<0.001)	OS: NR vs. NR (HR 0.60, p=0.001) ORR: 55.7% vs. 27.1% (p<0.001)
CLEAR trial (15)	1st line	Lenvatinib (L) + Pembrolizumab (P) vs. Lenvatinib + Everolimus (E) vs. Sunitinib (S)	ccRCC	n=1069 (355/357/357)	PFS (L+P vs. S): 23.9 vs. 9.2m (HR 0.39, p<0.001) PFS (L+E vs. S): 14.7m vs. 9.2m (HR 0.65, p<0.001)	OS (L+P vs. S): NR vs. NR (HR 0.66, p=0.005) OS (L+E vs. S): NR vs. NR (HR 1.15, p=0.30)
Keynote-564 (16)	Adjuvant therapy	Pembrolizumab vs. Placebo	ccRCC	n=994 (496/498) Intermediate risk (427/433)^†^ High risk (40/36)^†^ M1 NED (29/29)^†^	DFS: 12m rate 85.7% vs. 76.2% 24m rate 77.3% vs. 68.1% (HR 0.68, p=0.0010)	OS: (HR 0.54, p=0.0164 not significant)
**Urothelial cancer**						
Javelin Bladder 100 (17)	Maintenance after 1st line (platinum doublet)	Avelumab vs. Best Supportive Care	Urothelial carcinoma	n=700 (350/350) PD-L1+ tumor 358 (189/169) Upper tract (106/81) Lower tract (244/269)	OS in overall population: 21.4m vs. 14.3m (HR 0.69, p=0.001) OS in PD-L1+tumor: NE vs. 17.1m (HR 0.56, p<0.001)	PFS in overall population: 3.7m vs. 2.0m (HR 0.62) PFS in PD-L1+ tumor: 5.7m vs. 2.1m (HR 0.56)
EV-301 trial (8)	3rd line	Enfortumab Vedotin vs. Docetaxel/Paclitaxel/Vinflunine	Urothelial carcinoma	n = 608 (301/307)	OS: 12.88m vs. 8.97m (HR 0.70, p=0.001)	PFS: 5.55m vs. 3.71m (HR 0.62, p<0.001)
Checkmate274 (18)	Adjuvant therapy	Nivolumab vs. Placebo	Muscle invasive urothelial carcinoma	n = 709 (353/356) Urinary bladder (279/281) Renal pelvis (44/52) Ureter (30/23)	DFS in ITT population: DFS at 6m: 74.9% vs. 60.3% DFS at 12m: 62.8% vs 46.6% (HR 0.70, p<0.001) DFS in tumors positive for PD-L1: DFS at 6m: 74.5% vs. 55.7% DFS at 12m: 67.2% vs 45.9% (HR 0.55, p<0.001)	Survival free from recurrence outside the urothelial tract: 40.5m vs. 29.5m alive and free from distant metastasis at 6m (ITT population): 82.5% vs. 69.8% (HR for distant metastasis or death 0.75) alive and free from distant metastasis at 6m (PD-L1+ population): 78.7% vs. 65.7% (HR for distant metastasis or death 0.61)
**Prostate cancer**						
Keynote-12, 28, 16, 158, 164 (19)	KN-12: ≥1 prior regimen KN-28: ≥1 prior regimen KN-16: -CRC: ≥2 prior regimens -non CRC: ≥1 prior regimen KN-158: ≥1 prior regimen KN-164: Prior FP, oxaliplatin, and irinotecan ± anti-VEGF/EGFR	Pembrolizumab (not randomized)	Solid tumor	KN-12: n = 6 KN-28: n = 5 KN-16: n = 30 (non CRC) KN-158: n = 19 KN-164: n = 61	ORR: 39.6% with a 7% CR Duration of response: from 1.6+ to 22.7+ m with 78% of responses lasting ≥6 m	–
PROfound trial (6)	After ARAT	Olaparib vs. another ARAT	mCRPC	n = 632 (256/131) Cohort A (n=162/83) had at least one alteration in BRCA1, BRCA2, or ATM	Imaging-based PFS in cohort A: 7.4m vs. 3.6m (HR 0.34, p<0.001)	Imaging-based PFS in the overall population: 5.8m vs. 3.5m (HR 0.49, p<0.001)

IMDC, International Metastatic renal cell cancer Database Consortium​; ccRCC, clear cell renal cell cancer; Fav., favorable; Int., Intermediate; OS, Overall survival; PFS, Progression free survival; ORR, Objective response rate; NR, Not reached; NE, could not be estimated; ITT, Intention-to-treat; DFS, Disease free survival; CRC, Colorectal cancer; mCRPC, metastatic castration resistant prostate carcinoma; ARAT, androgen receptor axis targeted agent.

† intermediate risk: pT2, grade4 or sarcomatoid, N0, M0; or pT3, any grade, N0M0, high risk: pT4, any grade, N0, M0; pT any stage, N+, M0, M1 NED: No evidence of disease after primary tumor + soft tissue metastases completely resected ≤ 1year from nephrectomy.

## Renal Cell Cancer

Angiogenesis inhibitors, which include sorafenib (Nexavar, Bayer), sunitinib (Sutent, Pfizer), bevacizumab (Avastin, Genentech/Roche), pazopanib (Votrient, Novartis), and axitinib (Inlyta, Pfizer) (20–24), plus two mechanistic target of rapamycin (mTOR) inhibitors, temsirolimus (Torisel, Pfizer) and everolimus (Affinitor, Novartis) (25, 26), are all currently available as a result of the first breakthrough in the medical treatment of metastatic RCC, although bevacizumab is not available in Japan. Nivolumab (Optivo, Ono Pharma/Bristol Myers Squib), which is a fully human IgG4 PD-1 antibody, selectively inhibits the interaction between PD-1 and both PD-L1 and PD-L2 (27). Its promising anti-tumor efficacy and manageable safety profile were demonstrated in the phase III Checkmate025 trial (27). Nivolumab therapy is thus being rapidly introduced in metastatic RCC clinical practice in Japan. Currently, TKIs and ICIs are the two main therapeutic agents in RCC medical therapy, and combined ICIs (nivolumab and ipilimumab [Yervoy, Bristol Myers Squib]) as well as combinations of an ICI and a TKI (pembrolizumab [Keytruda, MSD] plus axitinib, and avelumab [Bavencio, Merck] plus axitinib) are mainstream as the first-line therapy for metastatic RCC (9–11). These can be considered as the second breakthrough caused by the ICIs. In addition, cabozantinib (Cabometyx, Takeda Pharmaceutical Company), which is a new-generation multi-kinase inhibitor that inhibits VEGFR as well as the receptor tyrosine kinases, MET and AXL, has been just approved for its superiority to everolimus as second-line treatment for the metastatic RCC in the phase III METEOR trial (12). As a first-line agent, cabozantinib also demonstrated better efficacy than sunitinib in the phase II CABOSUN trial (13). Adding to these therapies, the combination of nivolumab plus cabozantinib and of pembrolizumab plus lenvatinib (Lenvima, Eisai Company) have been just launched in Japanese clinical practice (14, 15). The first-line therapy is likely to be a mixture of the various combination therapies. In addition, the adjuvant pembrolizumab may become a standard of care for patients with high-risk non-metastatic RCC after nephrectomy or partial nephrectomy (16).

### Nivolumab Plus Ipilimumab, Pembrolizumab Plus Axitinib, and Avelumab Plus Axitinib

As noted above, the combined ICIs (nivolumab and ipilimumab) and the combinations of an ICI and a TKI (pembrolizumab plus axitinib, avelumab plus axitinib) are currently mainstream as first-line therapy for metastatic RCC (10–12). These combinations all demonstrated superior efficacy to sunitinib with a tolerable safety profile in the phase III CheckMate-214, Keynote-426, and Javelin Renal 101 clinical trials, respectively (9–11)

Currently, there is no validated recommendation to select the first-line therapy among these three regimens although the combination of nivolumab and ipilimumab is approved only for the International mRCC Database Consortium (IMDC) intermediate/poor category. Numerically, nivolumab plus ipilimumab had a higher complete response (CR) rate (9%) than the ICI plus TKI regimens (5.8% for pembrolizumab and axitinib and 4.4% for avelumab plus axitinib) (9–11). In the sub-analysis of CheckMate-214, which consisted of 139 patients with intermediate- and poor-risk sarcomatoid RCC (28), nivolumab plus ipilimumab compared to sunitinib demonstrated a higher median overall survival (OS; not reached vs 14.2 months), progression-free survival (PFS; 26.5 months vs 5.1 months), and objective response rate (ORR; 60.8% vs 23.1%) (28). It is particularly noteworthy that this combination achieved the highest CR rates ever (19%) for metastatic RCC patients with sarcomatoid component (28). On the other hand, in this combination therapy, 28% of patients either had progressive disease (PD) as the best response or were not evaluable, compared to ~16% for pembrolizumab plus axitinib and ~15% for avelumab plus axitinib (9–11). These are smaller percentages than with nivolumab plus ipilimumab therapy. When prompt disease control is necessary due to rapidly symptomatic progressive disease, a combination of ICI and TKI may be the preferred strategy (29, 30).

### Cabozantinib

The METERO trial is the randomized, open-label, phase III clinical trial (n = 658) that compared the efficacy of cabozantinib with everolimus in patients with metastatic RCC who had progressed after TKI therapy (12). The median PFS (7.4 vs 3.8 months, HR 0.58, *P* < 0.001), OS (HR 0.67, *P* = 0.005), and ORR (21% vs 5%, *P* < 0.001) were higher for patients treated with cabozantinib than everolimus (12). In addition, the CABOSUN trial is a randomized phase II multicenter trial that compared cabozantinib with sunitinib as first-line therapy in patients with treatment-naïve metastatic RCC (n = 157, IMDC intermediate/poor category) (13). The median PFS (8.2 vs 5.6 months, HR 0.66, *P* = 0.012), ORR (33% vs 12%), and disease control rate (DCR: 75% vs 47%) were higher for patients treated with cabozantinib than sunitinib (13). Cabozantinib is the first agent that demonstrated greater efficacy than sunitinib for the treatment-naïve metastatic RCC patients. Therefore, cabozantinib is currently considered as one of the standard options for second-line treatment after not only the ICI/TKI but also the ICI/ICI combination therapies, and as an alternative first-line therapy for those patients who are ineligible for ICI therapy.

### Nivolumab Plus Cabozantinib

The Checkmate 9ER is the phase III open-label randomized clinical trial (n=651) for previously untreated metastatic RCC (28). The median PFS, the probability OS at 12 months, and the ORR of the patients treated with nivolumab plus cabozantinib vs sunitinib were 16.6 months vs 8.3 months (HR 0.51, *P* < 0.001), 85.7% vs 75.6% (HR 0.60, *P* = 0.001), and 55.7% vs 27.1% (*P* < 0.001), respectively (14). Grade 3 or higher AEs for any cause were 75.3% for nivolumab plus cabozantinib vs 70.6% for sunitinib (14). In addition, patients reported better health-related quality of life with nivolumab plus cabozantinib than with sunitinib (14).

### Pembrolizumab Plus Lenvatinib

Lenvatinib, another anti-angiogenesis agent, acts as a multiple kinase inhibitor against the VEGFR1, VEGFR2, and VEGFR3 (15). The combination of lenvatinib and pembrolizumab demonstrated superior efficacy to sunitinib in the phase III international clinical (the CLEAR) trial (15). In this trial, a total of 1069 patients were randomly assigned to receive lenvatinib plus pembrolizumab (n = 355), lenvatinib plus everolimus (n = 357), or sunitinib (n = 357) (15). The median PFS and OS periods were longer with lenvatinib plus pembrolizumab than with sunitinib (median PFS: 23.9 vs 9.2 months, HR 0.39, *P* < 0.001, OS: HR 0.66, *P* = 0.005, respectively) (15). Grade 3 or higher adverse events emerged or worsened during treatment in 82.4% of the patients who received lenvatinib plus pembrolizumab; those in at least 10% of the patients in this group included hypertension, diarrhea, and elevated lipase levels (15).

Nivolumab plus cabozantinib and pembrolizumab plus lenvatinib are the third and fourth ICI-plus-TKI combination therapies for metastatic RCC.

### Adjuvant Pembrolizumab After Radical Surgery

Keynote-564 is a phase III, double-blind, multicenter trial of pembrolizumab vs placebo following nephrectomy in patients with high-risk clear cell RCC (16). High-risk criteria included pT3, pT4, or any N+ disease. In the pT2 cases, tumors with Fuhrman Grade 4 or sarcomatoid component included were considered to be high-risk (16). The high-risk category also included having no evidence of disease after resection of oligometastatic sites ≤ 1 year from nephrectomy. Adjuvant pembrolizumab after nephrectomy demonstrated a statistically significant increase in disease free survival (DFS: primary endpoint) compared with placebo (HR 0.68, *P* = 0.001) (16). The DFS rates for pembrolizumab vs placebo were 85.7% vs 76.2% at 12 months and 77.3% vs 68.1% at 24 months (16). Safety results were in line with expectations, and there was low incidence (7.4%) of high-dose corticosteroid treatment for immune-related AEs (16). The investigators concluded that adjuvant pembrolizumab will be a potential new standard of care for the patients with high-risk RCC after the radical surgery, although additional follow-up is planned for the key secondary endpoint of OS (16).

### In 2022, the First-Line Combination Era for Metastatic Renal Cell Cancer (RCC)

**Figure 1:**
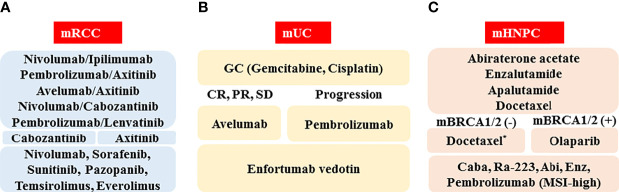
Schematic of standard of care in 2022 for medical treatment of metastatic urological cancers, including renal cell cancer (mRCC, **A**), urothelial cancer (mUC, **B**), and hormone naïve prostate cancer (mHNPC, **C**). Abbreviations: CR: complete response; PR: partial response, SD: stable disease; mBRCA(-): BRCA1/2 mutation negative; mBRCA(+): BRCA1/2 mutation positive; Caba: cabazitaxel; Abi: abiraterone acetate; Enz: enzalutamide; MSI-high: microsatellite instability-high. *When docetaxel is administered as the first-line therapy for metastatic hormone-sensitive prostate cancer (HSPC), abiraterone acetate, enzalutamide, cabazitaxel, Ra-223, pembrolizumab (MSI-high), and olaparib (mBRCA[+]) are the candidates for metastatic castration-resistant prostate cancer (CRPC).

The schematic standard of care for medical treatment of metastatic RCC in 2022 is depicted in **Figure 1A**. As first-line therapy, there are five optional combination therapies (nivolumab plus ipilimumab, pembrolizumab plus axitinib, avelumab plus axitinib, nivolumab plus cabozantinib, and pembrolizumab plus lenvatinib; however, nivolumab plus ipilimumab therapy is approved only for the IMDC intermediate/poor category). Because there is currently no validated recommendation to select first-line therapy, biomarkers to predict the response and prognosis are vitally important. As second-line therapy, cabozantinib and axitinib will often be chosen following pembrolizumab plus axitinib, avelumab plus axitinib, or nivolumab plus cabozantinib. For the patients treated with nivolumab plus ipilimumab or pembrolizumab plus lenvatinib, both cabozantinib and axitinib are candidates. The representative clinical trials for metastatic RCC are shown in **Table 2**.

**Table 2:** Ongoing clinical trials of the medical treatment for the urological cancers.

Trial/NCT No.	Study design	Treatment	Cancer type	Patients	Primary endpoint
**Renal cell cancer**					
PIVOT-09/ NCT03729245	Phase III, randomized, open-label study	Bempegaldesleukin (NKTR-214: BEMPEG) in combination with Nivolumab compared with the investigator’s choice of a TKI therapy (either Sunitinib or Cabozantinib monotherapy)	Advanced metastatic RCC	n=623 (actual)	ORR using mRECIST 1.1 by BICR in IMDC intermediate- or poor-risk patients; ORR per mRECIST 1.1 by BICR in IMDC all-risk patients; OS in IMDC intermediate- or poor-risk patients; OS in IMDC all-risk patients
PDIGREE/ NCT03793166	Phase III, randomized, open-label study	Nivolumab and Ipilimumab followed by Nivolumab vs. Cabozantinib with Nivolumab	Metastatic untreated RCC	n=1046 (estimated)	OS
COSMIC-313/ NCT03937219	Phase III, randomized, double-blind, controlled study	Cabozantinib with Nivolumab and Ipilimumab vs. Nivolumab and Ipilimumab	Previously untreated advanced or metastatic RCC of intermediate or poor risk	n=840 (estimated)	Duration of PFS per RECIST 1.1 as determined by BIRC
CONTACT-03/ NCT04338269	Phase III, multicenter, randomized, open-label study	Atezolizumab + Cabozantinib vs. Cabozantinib alone	Advanced RCC	n=500 (estimated)	PFS as assessed by IRF; OS
MK-6482-005/ NCT04195750	Phase III, open-label, randomized study	Belzutifan (MK-6482)*^1^ vs. Everolimus *^1^ Belzutifan (MK-6482): a potent and selective small molecule inhibitor of HIF-2α	Advanced RCC	n=736 (estimated)	PFS per RECIST 1.1; OS
MK-6482-011/ NCT04586231	Phase III, open-label, randomized study	MK-6482 + Lenvatinib (MK-7902) vs. Cabozantinib [2^nd^-line or 3^rd^-line treatment]	Advanced RCC	n=708 (estimated)	PFS per RECIST 1.1 as assessed by BICR, OS
MK-6482-012/ NCT04736706	Phase III, open-label, randomized study	Pembrolizumab (MK-3475) + Belzutifan (MK-6482) and Lenvatinib (MK-7902), or Pembrolizumab/Quavonlimab (MK-1308A) + Lenvatinib vs. Pembrolizumab and Lenvatinib [1^st^-line treatment]	Advanced ccRCC	n=1431 (estimated)	PFS according to RECIST 1.1 as assessed by BICR; OS
**Urothelial cancer**					
CheckMate 274/ NCT02632409 (1)	Phase III, randomized, double-blind, multi-center study	adjuvant Nivolumab vs. placebo (following surgery to remove the cancer)	High risk invasive UC	n=709 (nivolumab n=353, placebo n=356)	DFS
AMBASSADOR/ NCT03244384	Phase III, randomized adjuvant study	MK-3475 (Pembrolizumab) vs. observation	MIBC and locally advanced UC	n=739 (estimated)	OS; DFS
KEYNOTE-905/ EV-303/ NCT03924895 (2)	Phase III, randomized study	cystectomy with perioperative Pembrolizumab and cystectomy with perioperative Enfortumab Vedotin and Pembrolizumab vs. cystectomy alone	CDDP-ineligible MIBC	n=836 (estimated)	pCR rate; EFS (in all pts, in pts whose tumors express PD-L1 CPS ≥10)
IMvigor010/ NCT02450331 (3)	Phase III, open-label, multicenter, randomized study	Atezolizumab vs. observation [adjuvant therapy]	High-risk MIUC after surgical resection	n=809 (actual)	DFS
KEYNOTE-866/ NCT03924856	Phase III, randomized, double-blind study	Perioperative Pembrolizumab (MK-3475) + NAC vs. perioperative placebo + NAC	CDDP-eligible MIBC	n=870 (estimated)	pCR rate; EFS
ONO-4538-86/ CA017078/ NCT03661320	Phase III, randomized study	NAC alone vs. NAC + Nivolumab or Nivolumab and BMS-986205*^2^, followed by continued post-surgery therapy with Nivolumab or Nivolumab and BMS-986205 *^2^ BMS-986205 (Linrodostat): an irreversible inhibitor of IDO1	MIBC	n=1200 (estimated)	pCR rate; EFS
NIAGARA/ NCT03732677	Phase III, randomized, open-label, multi-center, global study	Durvalumab + GEM/CDDP for neoadjuvant treatment followed by Durvalumab alone for adjuvant treatment	Bladder cancer	n*=*1050 (estimated)	pCR rates at time of cystectomy; EFS per central review defined as time from randomization to event
DANUBE/ NCT02516241 (4)	Phase III, randomized, open-Label, controlled, multi-center, global study	first-Line MEDI4736 (Durvalumab) monotherapy and MEDI4736 (Durvalumab) + Tremelimumab vs. SoC CTx	Unresectable Stage IV UC	n=1126 (actual)	To assess the efficacy of Durvalumab + Tremelimumab combination therapy vs. SoC in terms of OS in FAS; To assess the efficacy of Durvalumab monotherapy vs. SoC in terms of OS in PD-L1-high analysis set
KEYNOTE-361/ NCT02853305 (5)	Phase III, randomized, controlled clinical trial	Pembrolizumab with or without platinum-based combination CTx vs. CTx	Advanced or metastatic UC	n=1010 (actual)	[Pembro Combo vs. CTx]: PFS using RECIST 1.1 as assessed by BICR; OS [Pembro vs CTx]: OS in pts with PD-L1 CPS ≥10%; OS
CheckMate 901/ NCT03036098	Phase III, open-label, randomized study	Nivolumab combined with Ipilimumab or with SoC CTx vs. SoC CTx	Previously untreated unresectable or metastatic UC	n=1290 (estimated)	OS in CDDP-ineligible randomized pts; OS in PD-L1 positive (≥1%) randomized pts by IHC; PFS by BICR; OS in CDDP-eligible pts with previously untreated; unresectable or metastatic UC
IMvigor130/ NCT02807636 (6)	Phase III, multicenter, randomized, placebo-controlled study	Atezolizumab as monotherapy and in combination with platinum-based CTx	Untreated locally advanced or metastatic UC	n=1200 (estimated)	PFS assessed by Investigator using RECIST 1.1 in pts treated with Atezolizumab combination therapy compared with placebo arm; OS; percentage of pts with AEs assessed using NCI-CTCAE v4.0
NILE/ NCT03682068	Phase III, randomized, open-label, controlled, multi-center, global study	combining Durvalumab ± Tremelimumab with SoC CTx (CDDP + GEM or CBDCA + GEM doublet) followed by Durvalumab monotherapy vs. SoC alone as first-line CTx	Metastatic bladder cancer	n=1434 (estimated)	OS
LEAP-011/ NCT03898180	Phase III, randomized, double-blind study	Pembrolizumab + Lenvatinib vs. Pembrolizumab + placebo	Advanced/unresectable or mUC	n=694 (estimated)	PFS per RECIST 1.1 as assessed by BICR; OS
EV-302/ NCT04223856	Phase III, open-label, randomized, controlled study	Enfortumab Vedotin + Pembrolizumab vs. SoC GEM + platinum-containing CTx	Previously untreated locally advanced or metastatic UC	n=760 (estimated)	Duration of PFS per RECIST 1.1 by BICR; Duration of OS
CREST/ NCT04165317	Phase III, multinational, randomized, open-label, three parallel-arm study	PF-06801591*^3^ + Bacillus Calmette-Guerin (BCG induction with or without BCG maintenance) vs. BCG (induction and maintenance) *^3^ PF-06801591 (Sasanlimab): an anti-PD-1 antibody	High-risk, BCG-naïve NMIBC	n=999 (estimated)	EFS (Arm A compared to Arm C); EFS (Arm B compared to Arm C)
TROPiCS-04/ NCT04527991	Phase III, randomized, open-label study	Sacituzumab Govitecan (IMMU-132)*^4^ vs. treatment of physician’s choice *^4^ Sacituzumab Govitecan: a novel ADC combining the humanized RS7 antibody targeting Trop-2 coupled to a proprietary hydrolyzable linker	Metastatic or locally advanced unresectable UC	n=600 (estimated)	OS
THOR/ NCT03390504	Phase III, randomized, open-label study	Erdafitinib vs. Chemotherapy (Vinflunine or Docetaxel) or Pembrolizumab	Advanced urothelial cancer harboring selected fibroblast growth factor receptor (FGFR) aberrations who have progressed after 1 or 2 prior treatments, at least 1 of which includes an anti-programmed death ligand 1(PD-[L]1) agent (cohort 1) or 1 prior treatment not containing an anti-PD-(L) 1 agent (cohort 2)	n=631 (estimated)	OS
**Prostate cancer**					
BLC3001/ NCT03748641	Phase III, randomized, placebo-controlled, double-blind study	Niraparib + Abiraterone acetate and PSL vs. Abiraterone acetate and PSL	mCRPC	n=1000 (estimated)	[Cohort 1 and 3] rPFS
IPATential/NCT03072238	Phase III, randomized, double-blind, placebo-controlled, multicenter trial	Ipatasertib + Abiraterone + PSL vs. placebo + Abiraterone + PSL	mCRPC	n=1101 (actual)	Investigator-assessed rPFS per PCWG3 criteria (PTEN Loss population); Investigator-assessed rPFS per PCWG3 (ITT population)
ARASENS/ NCT02799602	Phase III, randomized, double-blind, placebo-controlled, multicenter study	Darolutamide (BAY 1841788/ODM-201) + standard ADT + Docetaxel vs. placebo + standard ADT + Docetaxel	mHSPC	n=1303 (actual)	OS
KEYLYNK-010/NCT03834519	Phase III, randomized open-label study	Pembrolizumab (MK-3475) + Olaparib vs. Abiraterone acetate or Enzalutamide	mCRPC	n=780 (estimated)	OS; rPFS per PCWG-modified RECIST 1.1 as assessed by BICR
CAPItello-281/ NCT04493853	Phase III, double-blind, randomized, placebo-controlled study	Capivasertib + Abiraterone vs. placebo + Abiraterone	*de novo* mHSPC by PTEN deficiency	n=1000 (estimated)	rPFS
TALAPRO-3/ NCT04821622	Phase III, randomized, double-blind study	Talazoparib + Enzalutamide vs. placebo + Enzalutamide	DDR gene muted mCSPC	n=550 (estimated)	Radiological PFS

ADC, antibody-drug conjugate; ADT, androgen deprivation therapy; AEs, adverse events; BCG, Bacille de Calmette et Guérin; BICR, blinded independent central review; BIRC, blinded independent radiology committee; CBDCA, carboplatin; ccRCC, clear cell renal cell carcinoma; CDDP, cisplatin; CPS, combined positive score; CRPC, castration-resistant prostate cancer; CTx, chemotherapy; DDR, DNA damage repair; DFS, disease-free survival; EFS, event-free survival; FAS, full analysis set; GEM, gemcitabine; HIF-2α, hypoxia-inducible factor 2α; IDO1, indoleamine 2,3-​dioxygenase 1; IHC, immunohistochemistry; IMDC, International Metastatic RCC Database Consortium; IRF, independent review facility; ITT, intention to treat; mCRPC, metastatic CRPC; mCSPC, metastatic castration-sensitive prostate cancer; mHSPC, metastatic hormone sensitive prostate cancer; MIBC, muscle-invasive bladder cancer; MIUC, muscle-invasive urothelial cancer; mRECIST, modified RECIST; mUC, metastatic UC; NAC, neoadjuvant chemotherapy; NCI-CTCAE v4.0, National Cancer Institute-Common Technology Criteria for Adverse Events version 4.0; NMIBC, non-muscle invasive bladder cancer; ORR, objective response rate; OS, overall survival; pCR, pathological complete response; PCWG, prostate cancer working group; PD-1, programmed cell death 1; PD-L1, programmed cell death ligand 1; PSL, prednisone/prednisolone; PTEN, phosphatase and tensin homolog deleted from chromosome 10; RCC, renal cell carcinoma; RECIST 1.1, response evaluation criteria in solid tumors version 1.1; rPFS, radiographic progression-free survival; SoC, standard of care; TKI, tyrosine kinase inhibitor; UC, urothelial cancer/carcinoma.

## Urothelial Cancer

Cytotoxic chemotherapy has long been the mainstay of medical therapy for metastatic UC. Currently, the gemcitabine plus cisplatin (GC) regimen is widely used throughout the world as standard first-line medical treatment (3). In 2017, pembrolizumab, which is a highly selective, humanized monoclonal IgG4κ isotype antibody against PD-1, was approved as the second-line treatment to be used after platinum-based chemotherapy. Based on its promising anti-tumor efficacy and manageable safety profile, pembrolizumab therapy is being rapidly introduced, and the paradigm of medical treatment for patients with metastatic UC has dramatically changed (3, 31). In addition, after first-line GC chemotherapy, maintenance therapy using avelumab, a fully human monoclonal antibody against PD-L1, has just launched in Japanese clinical practice with the excellent results of the phase III Javelin Bladder 100 clinical trial (17). Moreover, enfortumab vedotin, which is a newly designed ADC anti-cancer agent, has been just approved as a third-line standard medical therapy after GC and ICI therapies (8). Enfortumab vedotin is composed of a fully human monoclonal antibody against nectin-4, a type I transmembrane cell adhesion protein that is highly expressed in a number of epithelial cancers, including urothelial cancer, and monomethyl auristatin E, an anti-cancer agent that disrupts microtubule formation in cancer cells (8). The ADC is a new type of anti-cancer agent, and the linker plays the important role of attaching the monoclonal antibody to the cytotoxic agent (32, 33). The linker is stable in the bloodstream and releases the drug into the cells only after binding to the target. Consequently, the nectin-4 targeted delivery of monomethyl auristatin E results in cell-cycle arrest and apoptosis for urothelial cancer cells (8, 32, 33). Finally, regarding patients with non-metastatic high-risk muscle-invasive UC (MIUC), adjuvant nivolumab after radical surgery will be approved in the near future (18).

### Avelumab

Javelin Bladder 100 was a phase III open-label clinical trial for patients with unresectable locally advanced or metastatic urothelial cancer who did not have disease progression with first-line chemotherapy (four to six cycles of GC or gemcitabine plus carboplatin) to receive maintenance avelumab plus best supporting care (BSC) or BSC alone (17). The median OS and the OS at 1 year were significantly better when treated with the avelumab plus BSC than with BSC alone (21.4 months, 71.3% vs 14.3 months, 58.4%, HR 0.69, *P* = 0.001). The median PFS for avelumab plus BSC was also longer than that of BSC alone (3.7 vs 2.0 months, HR 0.62, 95% confidence interval [CI]: 0.52-0.75) (17). The incidence of adverse events of Grade 3 or higher was 47.4% with avelumab plus BSC vs 25.2% with BSC alone. Maintenance avelumab therapy is a current standard of care for patients who have responded to the first-line GC [CR, PR, or stable disease (SD)] (17).

### Enfortumab Vedotin

The EV-301 clinical trial is a global, open-label, randomized phase III clinical trial of enfortumab vedotin for the treatment of metastatic UC patients who had previously received both platinum-containing and ICI therapies. The control arm consisted of investigator-chosen chemotherapy (docetaxel, paclitaxel, or vinflunine) (8). The median PFS and OS for the enfortumab vedotin group (n = 301) were longer than for the chemotherapy group (n = 307) (PFS: 5.55 vs 3.71 months, HR: 0.62, *P* < 0.001; OS: 12.88 vs 8.97 months, HR: 0.70, *P* = 0.001). Regarding the safety profile, the incidence of events of Grade 3 or higher was similar in the two groups (51.4% and 49.8%, respectively) (8). Grade 3 or higher treatment-related adverse events that occurred in at least 5% of patients receiving enfortumab vedotin included maculopapular rash (7.4%), fatigue (6.4%), and decreased neutrophil count (6.1%) (8). Because of the excellent efficacy and the controllable safety profile, enfortumab vedotin represents an important novel therapeutic strategy as third-line therapy for patients who experienced both platinum-containing and immune checkpoint inhibitor therapies.

In addition, there are two agents, which were recently granted accelerated approval by the United States Food and Drug Administration (FDA). One is erdafitinib, which is a tyrosine kinase inhibitor of fibroblast growth factor receptor (FGFR) 1-4 (34). Erdafitinib demonstrated antitumor activity in an open-label, phase II study, which enrolled patients with metastatic UC (n=99), who had *FGFR* mutations (34). In this phase II study, the confirmed ORR, and the median PFS and OS periods were 40% (CR: 3%, PR: 37%), 5.5 months, 13.8 months, respectively (34) Among the 22 patients who had undergone previous immunotherapy, the ORR was 59% (34). Treatment-related AEs of grade 3 or higher were reported in 46% of the patients and almost all were managed by dose reduction (34). No treatment-related death was reported.

Another agent is sacituzumab govitecan. Sacituzumab govitecan is a new ADC and a monoclonal antibody specific for Trop-2 conjugated with SN-38, which is the active metabolite of irinotecan (35). The TROPHY-U-01 trial is a multicohort, open-label, phase II study. In this clinical trial, cohort 1 included patients (n=113) with locally advanced or unresectable or metastatic UC who had progressed after prior platinum-based combination chemotherapy and checkpoint inhibitors (35). At a median follow-up of 9.1 months, the ORR, the median PFS and the median OS periods were 27%, 5.4 months, and 10.9 months, respectively (35). Regarding grade ≥ 3 adverse events, neutropenia (35%), leukopenia (18%), anemia (14%), diarrhea (10%), and febrile neutropenia (10%) were seen and 6% discontinued due to treatment-related AEs (35). The respective confirmatory clinical trials are currently underway.

To date, various clinical trials using enfortumab vedotin as first-line therapy for metastatic urothelial cancer have been conducted. Among them, high response rates were reported using enfortumab vedotin in combination with pembrolizumab as first-line treatment for metastatic disease (8). The CR, OR, and DCR rates were 13%, 71%, and 93%, respectively. The phase III EV-302 clinical trial, which randomizes patients with treatment naive metastatic urothelial cancer to the combination of enfortumab vedotin and pembrolizumab or to the standard of care platinum-based chemotherapy, is ongoing (8, 32, 33).

### Adjuvant Nivolumab

CheckMate 274 is a phase III, randomized, double-blind, multicenter study of adjuvant nivolumab vs placebo in patients with high-risk MIUC (18). In this study, the high-risk patients were those with ypT2-ypT4a or ypN+ MIUC who had neoadjuvant cisplatin chemotherapy and those with pT3-pT4a or pN+ MIUC without prior neoadjuvant cisplatin chemotherapy. The planned therapy started within 120 days after radical surgery. Primary endpoints were DFS in the intent-to-treat (ITT) population and DFS in the patients with tumor PD-L1 ≥ 1%. Stratification factors were PD-L1 status (<1% vs ≥ 1%), presence and absence of prior neoadjuvant cisplatin-based chemotherapy, and nodal status (+ vs -). Adjuvant therapy was performed for up to 1 year. Among the ITT population, the median DFS period of the nivolumab group was significantly longer than that of the placebo group (21.0 vs 10.9 months, HR: 0.70, *P* < 0.001) (18). Regarding the PD-L1 ≥ 1% patients, the median DFS for the nivolumab group was also longer (not reached vs 10.8 months, HR: 0.53, *P <*0.001). The safety and tolerability of nivolumab monotherapy was consistent with previous reports in other tumor types, including in patients with metastatic UC (18). In addition, no deterioration in health-related quality of life was observed with nivolumab vs placebo (18). Based on these excellent results, approval of adjuvant nivolumab after radical surgery is anticipated in clinical practice.

### In 2022, GC Chemotherapy, Pembrolizumab, and Enfortumab Vedotin as the Respective Standard First-, Second, and Third-Line Medical Therapy for Metastatic Urothelial Cancer (UC)

A schematic standard of care for treatment of metastatic UC in 2022 is shown in **Figure 1B**. The GC regimen, pembrolizumab, and enfortumab vedotin are recommended as the first-, the second-, and the third-line agents, respectively. Because there are various ongoing clinical trials that can reveal the next generation standard of care (**Table 2**), the standard medical treatment of the metastatic UC has been and will continue to be changing year by year.

## Prostate Cancer

Hormonal therapy, which includes androgen deprivation therapy with or without androgen receptor axis-targeted (ARAT) agents, has been the mainstay in the medical treatment for metastatic and non-metastatic prostate cancer. Apart from bicalutamide and flutamide, which are often referred to as “vintage hormones,” docetaxel was previously the only agent approved for prolonging the survival of castration-resistant prostate cancer (CRPC) patients in Japan (36, 37). Currently, however, several effective systemic agents are available to these patients in Japanese clinical practice, including the new ARAT agents, enzalutamide (Xtandi, Astellas), abiraterone acetate (Zytiga, Jansen Pharmaceutical K.K.), apalutamide (Earleada, Jansen Pharmaceutical K.K.), and darolutamide (Nubeqa, Beyer HealthCare); an alpha emitter, radium-223 dichloride (Xofigo, Beyer HealthCare); and the novel taxane chemotherapy agent, cabazitaxel (Jevtana, Sanofi) (38–43),. In addition, abiraterone, apalutamide, and enzalutamide are approved for the metastatic hormone-sensitive prostate cancer (HSPC) treatment (44–46). Due to their excellent efficacy and manageable toxicity, these agents are rapidly being introduced into clinical practice in Japan, dramatically changing the therapeutic strategy for metastatic prostate cancer.

In 2018, pembrolizumab (Keytruda, MSD) was approved for the treatment of metastatic solid tumors including prostate cancer in patients with microsatellite instability (MSI)-high disease (19, 47, 48). In addition, 2021 saw the launch in Japanese clinical practice of olaparib (Lynparza, AstraZeneca), which is a novel PARP inhibitor, for the treatment of metastatic CRPC with BRCA1/2 mutation (6). Despite coverage under the Japanese universal health insurance system, the efficacy and safety profile of these agents for CRPC patients have been poorly documented so far, probably due to its rarity.

### Pembrolizumab

The United States Food and Drug Administration (US FDA) approval of pembrolizumab for the treatment of metastatic solid tumors in patients with MSI-high was based on the excellent results in five single-arm clinical trials, Keynote-12, -28, -16, -158, and -164 (45, 46). The ORR was 39.6% (95% CI: 31.7-47.9) with a 7% CR rate among 149 heavily treated patients with 15 different tumor types, including a single CRPC patient (19). The duration of response ranged from 1.6 to 22.7 months, with 78% of responses lasting ≥ 6 months (19). The adverse event profiles of pembrolizumab were similar to those observed across prior trials in other indications (19). This approval is the first time that the FDA has approved a cancer treatment for an indication based on a common biomarker, regardless of the primary site. Previously, we reported a first Japanese CRPC case that demonstrated clinical benefit from pembrolizumab treatment (47). The rarity of MSI-high tumors in CRPC may hamper pembrolizumab administration. This potentially active agent, however, should be considered as part of a treatment regimen for patients with MSI-high CRPC.

### Olaparib

The PROfound trial is a prospective phase III trial for the patients with metastatic CRPC who had disease progression while receiving an ARAT agent (enzalutamide or abiraterone) (6). All patients had a qualifying alteration in prespecified genes with a direct or indirect role in homologous recombination repair (HRR) and were randomly assigned to receive the PARP inhibitor olaparib or the ARAT agents enzalutamide or abiraterone (control group) (6). The median radiological PFS in patients with alterations in *BRCA1*, *BRCA2*, or *ATM* (the primary endpoint) was significantly longer in the olaparib group than in the control group (7.4 vs 3.6 months, HR: 0.34, *P* < 0.001) (47). A significant benefit was also observed with respect to the confirmed ORR (33% vs 2%, HR: 20.86, *P* < 0.001) and the time to pain progression (HR: 0.44, *P* = 0.02) (6). Although 81% of the patients in the control group who had progression crossed over to receive olaparib, the median OS in the patients with alterations in *BRCA1*, *BRCA2*, or *ATM* in the olaparib group was longer than in the control group (18.5 vs 15.1 months, HR: 0.64, *P* = 0.02) (6). The safety profile in patients who received olaparib was manageable, and anemia and nausea were the main toxic effects (6). Because the exploratory analyses suggested that patients harboring *BRCA1* or *BRCA2* alterations derived the most benefit, olaparib was approved for the treatment of metastatic CRPC harboring BRCA1/2 mutation in Japan.

### In 2022, Androgen Deprivation Therapy (ADT) With New Androgen Receptor Axis-Targeted (ARAT) Agents as the Standard First-Line Era for the Metastatic Prostate Cancer

The schematic standard of care for the medical treatment of metastatic prostate cancer in 2022 is depicted in **Figure 1C**. As first-line therapy, there are three optional hormone therapies with ADT, which include abiraterone acetate, enzalutamide, and apalutamide, although an abiraterone acetate therapy is approved only for the LATITUDE high-risk category (46). In addition, due to the positive results of the CHAARTED trial, docetaxel therapy for patients with metastatic HSPC has just been formally approved in Japan (49). When progression during the first-line therapy is seen, it is necessary to confirm the presence or absence of a BRCA1/2 mutation. If a BRCA1/2 mutation is found, the patient should be treated with olaparib. If not, docetaxel chemotherapy should be considered for patients with a good performance status. As third-line or later therapies, the ARAT agents (including abiraterone acetate and enzalutamide), the chemotherapeutic agent cabazitaxel, radium-223, and pembrolizumab (MSI-high) are awaited.

## Future Directions

Due to vigorous medicine developments, the standard medical treatment of urological cancer has been and will be changing year by year. The major ongoing studies are summarized in **Table 2**. For the treatment of metastatic RCC, five combination therapies (nivolumab plus ipilimumab, pembrolizumab plus axitinib, avelumab plus axitinib, pembrolizumab plus lenvatinib, nivolumab plus cabozantinib) as described above were approved. The focus of the exploitation seems to be shifting to adjuvant therapy after radical nephrectomy (**Tables 1**, **2**). For the treatment of invasive urothelial cancer, various pre- and post-surgical clinical trials with radical cystectomy are being conducted. Besides the CheckMate274 described above, AMBASSADOR, Keynote-905/EV-303, and IMvigor010 are ongoing phase III trials using pembrolizumab, pembrolizumab plus enfortumab vedotin, and atezolizumab, respectively (**Table 2**) (50–54). For patients with metastatic UC, since Sternberg et al. in 1985 reported the excellent results of the cisplatin-based multi-agent chemotherapy regimen known as MVAC (methotrexate, vinblastine, Adriamycin, cisplatin), no medical treatment, including the GC regimen, has been more effective (3, 55). Currently, various ongoing clinical trials are using ICIs compared with GC, and the establishment of a brand-new first-line regimen for this disease is expected (**Table 2**). In addition, there are various ongoing trials for metastatic prostate cancer. Among them, the ARASENS trial is comparing ADT, docetaxel, and darolutamide with ADT and docetaxel for patients with metastatic HSPC (**Table 2**). For patients with metastatic CRPC, KeyLynk-010 is a study comparing ADT, olaparib plus pembrolizumab with ADT plus abiraterone or enzalutamide (**Table 2**). On the other hand, as discussed at the Advanced Prostate Cancer Consensus Conference (APCCC) 2019, we understand that the novel treatment of ^177^Lu-PSMA-617 radioligand therapy for metastatic prostate cancer is one of the most attractive candidates (56, 57). However, as it may take considerable time to introduce it into Japanese clinical practice, we did not cover it in this review. Finally, we have to remind ourselves of another important issue, genome information-based medical therapy. To date, somatic mutation is usually based on the examination of tissue removed by surgery or biopsy. However, in sampling for the PROfound trial, 30% of the biopsies were not suitable for DNA analysis (58). High concordance between tumor tissue and the circulating tumor DNA (ctDNA) has been reported, with 81% positive percentage agreement and 92% negative percentage agreement (59, 60). The FoundationOne Liquid has just been approved in Japan. Liquid biopsies may alter genome information-based medical therapy (58–60). These results, along with others, will be awaited with high expectations.

## Conclusion

In this review, we introduced agents and regimens that have just launched or will be launched in the near future in Japan. The efficacies and safety profiles are being or will be evaluated in Japanese clinical practice (**Table 1**). In addition, we summarized ongoing clinical trials (**Table 2**). At this time, various combination therapies, including ICI, cytotoxic chemotherapy, and new agents including novel ADCs are being investigated in clinical trials (**Table 2**). We await the results of these trials with high expectations for new therapies. Although we illustrated the predicted standards of care for metastatic urological cancer in **Figure 1**, the standards of care will be changing year by year.

## Author Contributions

TY conceived and designed the study. TY also supervised the manuscript. TY, TU, and RO contributed to acquisition, analysis, and interpretation of data, writing and critical revision of the final manuscript. All authors contributed to the article and approved the submitted version.

## Conflict of Interest

TY received remuneration for a lecture from Astellas (Tokyo, Japan), Sanofi Japan (Tokyo, Japan), Pfizer Japan (Tokyo, Japan), Novartis Pharma Japan (Tokyo, Japan), Ono Pharma (Osaka, Japan), Bristol-Myers Squibb Japan (Tokyo, Japan), MSD Japan (Tokyo, Japan), Jansen Pharmaceutical K.K. (Tokyo, Japan), and Merck (Tokyo, Japan).

The remaining authors declare that the research was conducted in the absence of any commercial or financial relationships that could be construed as a potential conflict of interest.

## Publisher’s Note

All claims expressed in this article are solely those of the authors and do not necessarily represent those of their affiliated organizations, or those of the publisher, the editors and the reviewers. Any product that may be evaluated in this article, or claim that may be made by its manufacturer, is not guaranteed or endorsed by the publisher.
